# Non-reliance of metazoans on stromatolite-forming microbial mats as a food resource

**DOI:** 10.1038/srep42614

**Published:** 2017-02-16

**Authors:** Gavin M. Rishworth, Renzo Perissinotto, Matthew S. Bird, Nadine A. Strydom, Nasreen Peer, Nelson A. F. Miranda, Jacqueline L. Raw

**Affiliations:** 1 DST/NRF Research Chair in Shallow Water Ecosystems, Nelson Mandela Metropolitan University, Port Elizabeth 6031, South Africa; 2 Department of Zoology, Nelson Mandela Metropolitan University, Port Elizabeth 6031, South Africa

## Abstract

Grazing and burrowing organisms usually homogenise microalgal mats that form on benthic sediments of many aquatic ecosystems. In the absence of this disruption, microalgal mats can accrete laminated deposits (stromatolites). Stromatolites are rare in modern coastal ecosystems, but persist at locations where metazoans are largely excluded. This study aimed to assess the trophic structure at stromatolite locations where metazoans co-occur, to determine the grazing influence exerted by the metazoans on the stromatolite-forming microalgae (cyanobacteria and diatoms). Stable isotope signatures (δ^13^C and δ^15^N) were used as food-web tracers and dietary composition of consumers was calculated using source mixing models. Results clearly demonstrate that the dominant macrofaunal grazers do not utilise stromatolite material as a food resource, but rather subsist on autochthonous macroalgae. For instance, the mean (±SD) dietary composition of two of the most abundant grazers, *Melita zeylanica* (Amphipoda) and *Composetia* cf. *keiskama* (Polychaeta), consisted of 80 ± 11% and 91 ± 7% macroalgae, respectively. This suggests that the stromatolite-forming benthic microalgae are not disrupted significantly by grazing pressures, allowing for the layered mineralisation process to perpetuate. Additionally, grazers likely have a restrictive influence on pool macroalgae, maintaining the competitive balance between micro- and macroalgal groups.

The shallow ocean floor during the Precambrian was a vastly different habitat to what it is today, with all areas then being dominated by extensive mats formed by microbial organisms^1^. Although these mats did remain into the Cambrian^2^, the rapid evolution of multicellular animals (metazoans) during this time (around 540 mya^3^) dramatically changed the marine landscape into one that was bioturbated^4^. Microbial mats that would once have formed laminated deposits (stromatolites) over time were then disrupted by metazoan activities. This is reflected in the fossil record, from as early as 3.5–3.7 bya^5^,^6^, whereby stromatolites are extensively-preserved prior to the Cambrian radiation of metazoan life^7^,^8^, but show a marked decrease following this period^9^ and are currently scarce in most modern environments.

Stromatolites are constructed by cyanobacteria that precipitate calcium carbonate as a by-product of metabolic activities, thereby forming layered accretions^10^,^11^. Other microalgae, such as diatoms, may further contribute to this layering process by trapping and binding sediment^12^. In modern circumstances where metazoans that graze and burrow are largely excluded, such as under hypersaline^13^ or highly erosive^14^ conditions, stromatolites may form. Indeed, experimental evidence has shown that the absence or removal of benthic fauna from microbial mats in shallow-water sediments results in distinct layered growth, which resembles stromatolites^15^. These observations on the apparent incompatibility of stromatolites and metazoans have resulted in the ‘metazoan-microbialite exclusion’ hypothesis^16^,^17^, which seeks to explain the Phanerozoic scarcity of stromatolites^9^, in addition to other limiting factors such as current, reduced ocean calcium carbonate levels^18^.

Perhaps counterintuitively, recent evidence has demonstrated that metazoans can and do coexist with living microbialites^19^, including distinctly-layered stromatolites^20^. The factors explaining this coexistence are complex. For example, in Cuatro Ciénegas, Mexico, oncolites (free-forming, spherical stromatolites) grow in a freshwater environment and co-occur with a high abundance of snails^21^,^22^. Within this habitat, the rapid growth rate of the microbialites, sustained by high concentrations of calcium carbonate, exceeds the rate of grazing by snails and thereby enable the stromatolites to persist^21^. Another hypothesis proposes that selective forces would favour the coexistence of metazoans with microbialites, due to the refugia benefits provided by the microbialite habitat. Benefits include increased oxygen resources at the benthic-water interface, as shown for past^1^,^23^ and modern^24^ microbialites, as well as escape from predators^19^,^22^.

In the peritidal stromatolites discovered in South Africa from the early 2000s^25^,^26^, a persistent assemblage of metazoans has been encountered^20^. These stromatolites grow in the upper intertidal to supratidal zone^25^ and are principally constructed by cyanobacteria and diatoms^27^. It seems that it is the dynamic and regularly-changing salinity conditions within the pools^28^ that drives the stromatolite community^27^,^29^, however the reasons why the benthic metazoan community is able to persist, without an apparently destructive influence on the stromatolites^20^, are as yet unclear. An understanding of the trophic dynamics within the pools would be particularly instructive in this regard. This would provide clarity on whether the coexisting metazoans are using the stromatolites as a food resource (grazing) or only as a habitat (burrowing^20^).

Therefore, the aim of this study was to investigate the food-web dynamics within the South African stromatolite pools. The key hypothesis being tested is that the infaunal metazoans directly inhabiting the stromatolite material, in addition to the epifaunal grazers, rely minimally on stromatolite microalgae as a food resource. Instead, the metazoans may rather consume other primary resources such as macroalgae, plant detritus or non-stromatolitic microalgae. This hypothesis is tested using a stable isotope (SI) approach (δ^13^C and δ^15^N)^30^,^31^,^32^ to infer the food resources consumed and the trophic level of each infaunal and epifaunal taxon within the stromatolite ecosystem. It has been shown that in other intertidal and estuarine habitats, benthic microalgae have a distinct carbon isotope signature compared to other producers (see Kang *et al*.^33^). This observation, if similarly apparent in the stromatolite sites, would enable stromatolite benthic microalgae to be distinguished amongst consumer organisms as a food resource. Although several studies have reported on the inorganic carbon component of stromatolite material^26^,^34^, this is the first study to investigate the organic carbon and trophic linkages in any extant stromatolite ecosystem.

## Results

### Physico-chemistry

Water within the barrage pool was stratified between benthic and surface layers, reflecting higher salinity and temperature in deeper water (Table 1). Cape Recife (site A) had noticeably lower nutrient concentrations, particularly for DIN, compared to the other sites, with all showing a decreasing trend from inlet seepage water to the ocean (Table 1). Benthic biomass was up to three orders of magnitude greater than pelagic biomass, with stromatolite benthic microalgae being comprised of cyanobacteria and diatoms in approximately equal proportions (Table 1).

### Food-web structure

**Figure 1:**
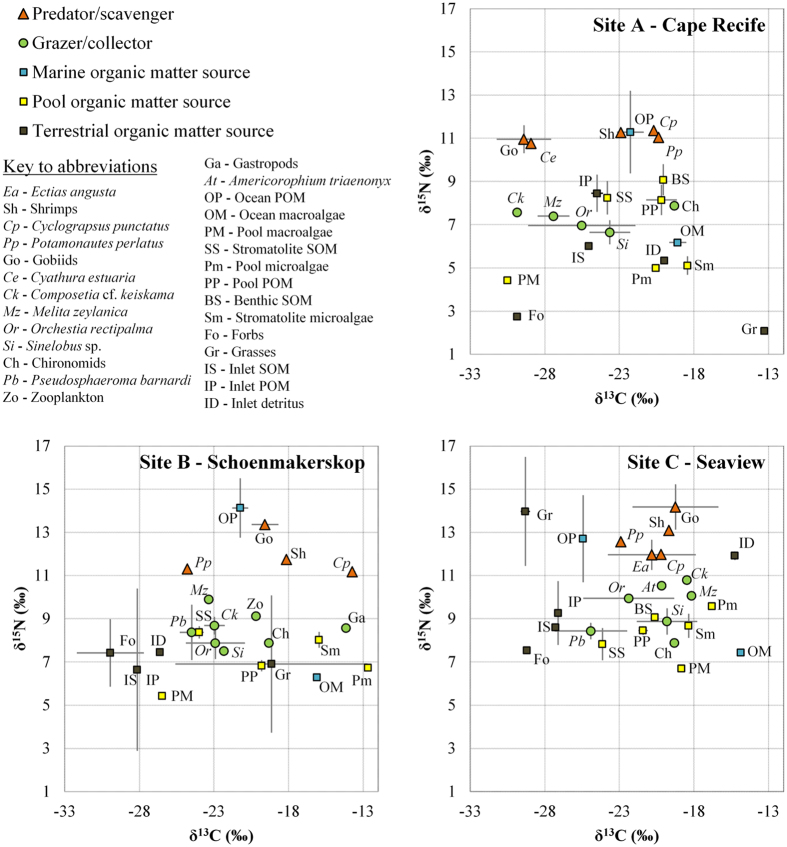
Mean (±SD) carbon and nitrogen stable isotope (‰) biplot of all measured components at the three sites along the South African coastline during August 2015. Epifaunal and infaunal samples are combined for respective species. Chironomid samples across the three sites were combined during processing to achieve a single, multi-site value.

There was a clear pattern of overall enrichment in the heavier N isotope between the three sites, with δ^15^N values (excluding inlet terrestrial and marine samples) ranging from 4.4 to 11.4, 5.4 to 13.4, and 6.7 to 14.9‰, for Cape Recife, Schoenmakerskop and Seaview, respectively (Fig. 1). δ^13^C signatures for these same stromatolite pool samples ranged from −12.7 to −24.5‰, with little evidence for any differences between sites, although Cape Recife samples generally had lower mean δ^13^C values (−24.5 ± 4.1‰) than both Schoenmakerskop and Seaview (−20.9 ± 4.0 and −20.5 ± 2.5‰, respectively). Terrestrial organic matter sources, apart from at Cape Recife, had lower δ^13^C signatures (mean: −25.1 ± 5.6‰; range: −13.3 to −31.5‰) than the fauna and flora in the stromatolite pools (Fig. 1). Additionally, marine organic matter sources were more enriched in the heavier ^13^C isotope (mean δ^13^C: −19.8 ± 4.0‰) compared to terrestrial sources. Marine POM was more enriched in ^15^N than most other samples collected and marine macroalgae were on the upper range of δ^13^C values recorded (Fig. 1). This suggests that the stromatolite food-web is largely supported by autochthonous organic matter sources, as consumer organisms and organic matter sources within the stromatolite pools (green circles and squares in Fig. 1) generally appeared closely associated with each other compared to marine and terrestrial inlet components.

In terms of trophic levels, there was a clear separation between predators or scavengers compared to grazers or collectors (Fig. 1), with the mean δ^15^N difference between these two levels across sites being 3.6 ± 0.3‰. Although there was some overlap between consumers and pool organic matter sources (Fig. 1), a consistent distinction between the two was also evident, with grazers and collectors being 1.1 ± 0.5‰ more enriched in the heavier ^15^N isotope than organic matter sources. At the top of the stromatolite pool food chain were the gobiids (*Coryogalops sordidus*), closely followed by the shrimps, *Palaemon peringueyi* (Fig. 1). The two brachyuran crabs (*Potomonautes perlatus* and *Cyclograpsus punctatus*) were similarly near the apex of the food-web, with *P. perlatus* having a lower δ^13^C signature than *C. punctatus* (−22.7 ± 2.2 versus −18.2 ± 3.9‰, respectively; Fig. 1). Also within the predator/scavenger guild, but only found at Cape Recife and Seaview, were the isopods *Cyathura estuaria* and *Ectias angusta*, respectively (Fig. 1). The dominant grazers/collectors were *Orchestia rectipalma* and *Melita zeylanica* (Amphipoda), *Sinelobus* sp. (Tanaidacea), and *Composetia* cf. *keiskama* (Polychaeta), all clustering around −23.2 ± 3.5‰ δ^13^C (Fig. 1). Other groups within this feeding guild were chironomid larvae, *Americorophium triaenonyx* (Amphipoda), *Pseudosphaeroma barnardi* (Isopoda), *Assiminea* cf. *capensis* (Gastropoda), and zooplankton (Fig. 1), but these were not consistent or abundant components across all sites. Of these, *P. barnardi* was most closely associated with the dominant grazers/collectors in terms of δ^13^C (mean: −24.7 ± 1.4‰), while the gastropods were most dissimilar (−14.1‰).

Isotopic niche space differed according to sampling sites, with Cape Recife reflecting the most and Schoenmakerskop the least variation amongst all samples collected (Table 2). Comparing amongst trophic guilds, organic matter sources that supported the stromatolite food chain occupied the widest isotopic niche space, reflecting approximately equal SEAc values amongst sites (Table 2). Grazers and collectors, as well as predators and scavengers, had similar isotopic niche widths. However, in general the grazer/collector guild was marginally broader, apart from at Schoenmakerskop (Table 2) where all four predator/scavenger species reflected unique isotope signatures, particularly for δ^13^C (Fig. 1), and therefore occupied a wide isotope niche space (SEAc = 18.1).

### Source and consumer comparisons

Organic matter sources within the stromatolite pools reflected a significant distinction between sediment, particulate and algal organic matter (Table 3). Stromatolite microalgae, as well as the microalgae growing within the pools but not forming stromatolite material, was both similar in terms of δ^13^C (p = 0.78; Table 3). The mean δ^13^C signature for pool macroalgae was substantially less enriched in the heavier ^13^C isotope compared to both microalgal groups (Table 3), this being particularly evident at Cape Recife and Schoenmakerskop, but not Seaview (Fig. 1). Particulate and benthic sediment organic matter were indistinguishable within the stromatolite pools (Table 3), as was also the case for these sources from the freshwater inlet (Fig. 1). The SOM within the stromatolite matrix was distinctly different in terms of δ^13^C to all other organic matter sources (Table 3), and also varied little between sites (Fig. 1). Similarly to the overall δ^13^C trend, pool organic matter sources were less enriched in the heavier ^13^C isotope at Cape Recife compared to the other two sites (Table 3).

**Figure 2:**
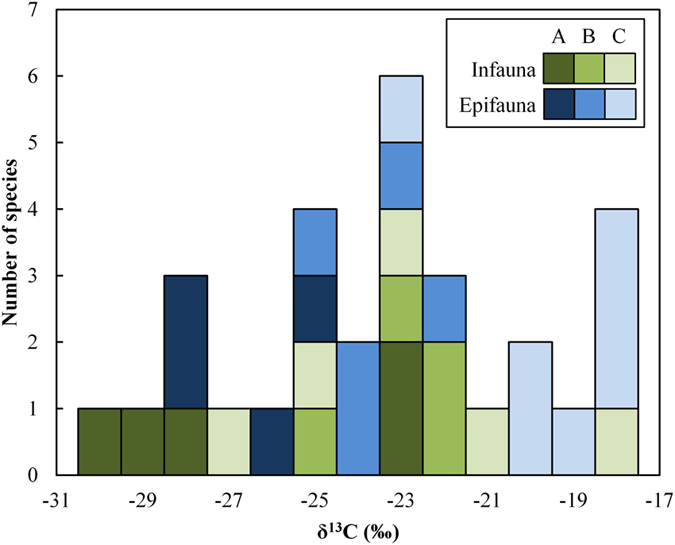
Frequency histogram of the δ^13^C signatures of all malacostracan and polychaete grazers/collectors (n = 31 samples) within the main pools at Cape Recife (**A**), Schoenmakerskop (**B**), and Seaview (**C**). Organisms are differentiated according to whether they are found directly within the stromatolite material (infauna: green bars) or sampled from within the pool waters (epifauna: blue bars).

There was no clear distinction between the δ^13^C of invertebrate infauna and epifauna (Fig. 2), although, on average, epifaunal samples were more enriched in the heavier ^13^C isotope than infaunal samples (mean: −22.7 ± 3.4 versus −24.2 ± 3.3‰, respectively). This difference was most noticeable at Seaview, with the other two sites reflecting unclear mixed signatures overall (Fig. 2). For those taxonomic groups found consistently within both the infauna and epifauna, namely the amphipods and isopods (n = 22 samples), the difference in terms of δ^13^C, when accounting for site-specific variability as a random intercept in the LMM model, was marginally non-significant (infauna respective to epifauna: C = −1.7 ± 1.0 SE; t = −1.6; p = 0.12). If the samples from Cape Recife were omitted from analysis, justified by the different C isotope profiles in terms of terrestrial inputs (Fig. 1) and pool organic matter sources (Table 2) between sites, then there was a significant difference between infauna and epifauna (n = 16 samples) for δ^13^C (infauna respective to epifauna: C = −2.5 ± 1.2 SE; t = −2.2; p < 0.05).

### Consumer diets

**Figure 3:**
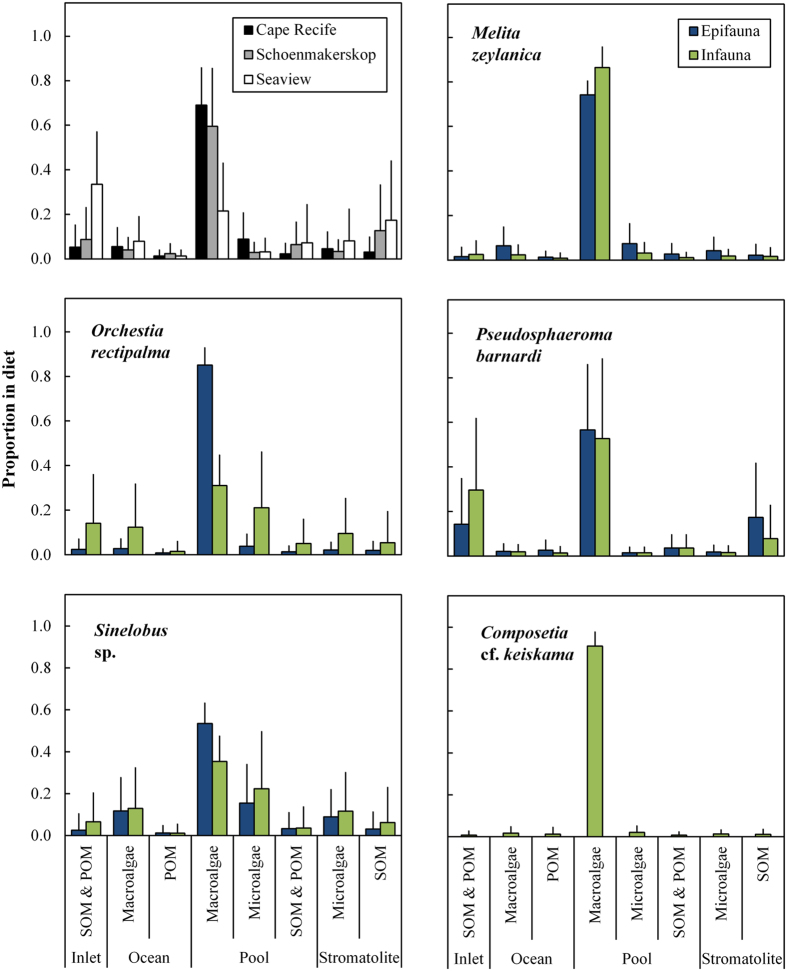
Relative proportion of organic matter sources (±SD) in the diets of dominant primary macrofaunal consumers, collected from stromatolite pools during August 2015. Diets were assessed using δ^13^C and δ^15^N isotopes as biotracers within a Bayesian mixing model, fitted using MixSIAR^62^. Proportions are expressed according to sampling sites and species, which were nested as hierarchical random factors within the mixing model. Species are reflected as infauna (green) and epifauna (blue) depending on their association with the stromatolite matrix. SOM (*sediment organic matter*); POM (*particulate organic matter*).

Within the consumer guild of dominant macrofauna occupying the stromatolite pools, consisting of *M. zeylanica, O. rectipalma* (both amphipods), *P. barnardi* (isopod), *Sinelobus* sp. (tanaid) and *Composetia* cf. *keiskama* (nereid polychaete), macroalgae associated with these pools were the primary dietary component overall according to the mixing model results (Fig. 3; overall mean: 26 ± 15% SD, Supplementary Table S1). This was especially apparent at sites A and B, with the macrofaunal consumers at site C ingesting a higher relative proportion of inlet organic matter compared to pool macroalgae (Fig. 3). Nereid polychaetes and *M. zeylanica* relied almost exclusively on pool macroalgae as a food source, while *O. rectipalma* and *Sinelobus* sp. had a generalist diet, but dominated by pool macroalgae and microalgae (Fig. 3). The isopod, *P. barnardi*, fed on a comparable amount of SOM and POM from inlet and stromatolite sources compared to pool macroalgae (Fig. 3), thereby accounting for the higher overall contribution of inlet sources to macrofaunal diet at site C mentioned previously. As for the frequency distribution of δ^13^C signatures (Fig. 2), there was no clear dietary distinction between infauna and epifauna food sources, with the most noticeable being *O. rectipalma* epifauna, which had a more exclusive diet on pool macroalgae compared to the generalist diet of infauna (Fig. 3). Overall, stromatolite-specific sources had a minimal contribution to the diets of all species, with stromatolite SOM reflecting a higher proportion in consumer isotopic signatures compared to stromatolite microalgae (Fig. 3; Supplementary Table S1). Similarly, marine sources (macroalgae and POM) were largely absent from consumer diets (Fig. 3; overall mean: 11 ± 10% SD and 7 ± 7% SD, respectively; Supplementary Table S1).

Organisms at the apex of the stromatolite pool food-web (Fig. 1) had generalist diets (Supplementary Table S1). Reflecting its ecology as a scavenger of animal material, the shrimp, *P. peringueyi*, favoured fish sources (mean dietary contribution from gobiids: 26 ± 13% SD) and otherwise consumed approximately equal proportions of available invertebrates (chironomids, gastropods, malacostracans and polychaetes; Supplementary Table S1). The omnivores associated with stromatolite pools (gobiids and brachyurans) demonstrated a higher proportion of primary organic matter components within their diets (individual source posterior distributions combined *a posteriori*^35^) from the stromatolite pools themselves compared to inlet or marine sources (Supplementary Table S1). Otherwise, gobiids (*C. sordidus*) fed on invertebrate sources, particularly shrimps, malacostracans and polychaetes (~11–12%; Supplementary Table S1). Both brachyuran species reflected small relative quantities of invertebrate sources, the highest of which were chironomids and malacostracans (~10%; Supplementary Table S1). The diet of *P. perlatus* comprised a greater proportion of inlet-associated material compared to *C. punctatus* (95% quantile: 1–59% versus 2–32%; Supplementary Table S1).

## Discussion

Stromatolites, which once dominated shallow oceans during the Precambrian^1^, are rare in modern coastal environments. This scarcity is attributed to the destructive impact of grazing and burrowing activities by multicellular organisms^9^, amongst other reasons. However, metazoans do coexist with extant stromatolites^19^,^20^, but little is known about the dynamics of this relationship. The results presented here therefore provide insight into how these unique structures might persist in modern peritidal environments, currently known from locations in South Africa, Western Australia and Northern Ireland.

### Grazing and stromatolite formation

The most revealing result from this study is the lack of any meaningful signature of stromatolite material or microalgae within the diets of the dominant grazer community, including those dwelling directly within the stromatolite matrix (Fig. 3). Instead, most macrofaunal invertebrate taxa consume macroalgae as a primary resource. This has two important implications from a stromatolite persistence perspective. Firstly, and directly, the microalgae that construct the stromatolite material^27^ are not consumed or disrupted through grazing activities, which allows for the layered depositional and mineralization process to continue unabated^10^,^11^ and thereby form stromatolites. Secondly, metazoan consumption of macroalgae associated with the stromatolite pools likely restricts macroalgal growth, thereby indirectly preventing the stromatolite microalgae from being outcompeted.

Should these grazing effects (or lack thereof) be different, the possible implications could be predicted based upon other comparable ecosystems. Grazing metazoans would usually be expected to disrupt and graze upon microalgal mats (e.g. ref. 33). Metazoan-grazer exclusion from estuarine microbial mats results in layered deposits that resemble stromatolites^15^. Subsequent reintroduction of metazoans then causes these microbial mats to revert to their usual, homogenised state. It seems from the current study that there are not extensive grazing pressures on the stromatolite material, otherwise the observed layering might be unexpected. Several hypotheses could explain why this might be the case. Although not quantified, pool macroalgae, which are dominated by *Ulva* spp^25^, may be more nutritious or palatable to the grazing metazoans than stromatolite microalgae. Preference for macroalgal species by macroinvertebrate grazers, depending on nutrient content, chemical defences or digestibility, have been observed in other intertidal environments^36^,^37^, although not necessarily consistently amongst ecosystems depending on the available producer species and consumer requirements^33^,^38^,^39^. Additionally, some authors have demonstrated the benefits provided to metazoans from microbialite habitats in terms of oxygen and predator refugia^22^,^23^,^24^. It therefore might be that selective forces are acting against destruction of the stromatolite matrix because of the direct micro-refugia benefit derived from this for metazoans, as outlined in Rishworth *et al*.^20^. Further dietary experimental work might elucidate this algal choice more clearly. However, in either respect, it is apparent from this study (Fig. 3) that metazoans do not exert a distinct grazing pressure on the stromatolite microfabric, and neither do the metazoans bioturbate the matrix when burrowing^20^. This has important implications from an historical perspective because of the view that both of these forces contributed towards the demise of the microbialites^8^,^9^,^16^.

The second major implication regarding the effect of metazoan grazing on the macroalgae can be highlighted in a study of another microbialite system. Steneck *et al*.^40^ showed how microbialites forming within the intertidal zone in the Bahamas are replaced by several types of macroalgae in areas where these macroalgae are not restricted by disturbance pressures. Stromatolite microalgae, and microalgae in general, are resistant to many types of ecological disturbances (such as inundation, hypersalinity or extreme temperatures) which would otherwise limit the occurrence of other algal groups^13^,^14^. It has been demonstrated at the South African stromatolite sites that frequent regime shifts in terms of salinity from freshwater to marine conditions, might be the driving force or pressure which excludes other organisms that would outcompete or disrupt the stromatolite microalgae^28^. In the stromatolite ecosystem of Shark Bay, Australia, hypersaline conditions are necessary to prevent the establishment of macroalgal taxa which would replace the stromatolite microalgae^13^. Results from the current study expand on this to suggest that metazoans may further contribute towards restricting the competitive advantage of macroalgae. Indeed, recent work at these sites indicates that there is an inverse correlation between metazoan abundance and macroalgal biomass (Rishworth *et al*., unpubl. data), which supports the grazing hypothesis presented here.

### The stromatolite food-web

Like estuaries, stromatolite pools receive resource inputs from both marine and freshwater material. Delineating the various contributions from these sources to consumer diets can be challenging, especially if the SI signatures of organic matter between allochthonous and autochthonous (in this case, the stromatolite pools) inputs are not distinct^41^. In this study, a trend of enriched δ^13^C values was observed in marine compared to freshwater sources. This pattern is similarly well-established in estuaries^42^. Pool organic matter sources were also consistently distinct between sampling sites, with stromatolite microalgae and SOM comparatively different to other autochthonous sources. These apparent distinctions allowed for a good identification of food sources in consumer diets, particularly at the primary consumer level. Across feeding guilds, autochthonous sources (pool macroalgae and microalgae, as well as sediment and particulate organic matter) were consistently the principal resource. Marine material (mostly macroalgae) was consumed in small quantities by macrofaunal invertebrates and also omnivorous gobiids and brachyurans, while terrestrial material was apparent in the diets of *Pseudosphaeroma barnardi* (isopod) and the two brachyurans. Expectedly, there was a greater proportion of inlet material in the SI signature of *Potomonautes perlatus* compared to *Cyclograpsus punctatus*, reflecting their known ecological preferences of freshwater versus intertidal habitats^25^. These two brachyurans also reflected more plant and algal material in their diets compared to the other omnivore assessed, *Coryogalops sordidus* (gobiid), although the results for all predators and omnivores were more equivocal than for the primary consumers.

Previous work suggests that the microalgal^27^,^29^ and infaunal (Rishworth *et al*., unpubl. data) community occupying the stromatolite pools is driven by bottom-up processes, including nutrient dynamics and physical forces such as temperature and salinity^28^. These conclusions have been reached because of the high primary producer biomass within the pools (which, as demonstrated here, support much of the stromatolite food-web) and the driving influence of physico-chemical characteristics. However, top-down forces in terms of grazing and predation are well-known from intertidal rocky shore habitats (as are those in which the stromatolite pools are found)^43^. Therefore, it might be expected that such processes are observed in the stromatolite pools. Indeed, as mentioned previously (see ‘Grazing and stromatolite formation’), top-down control of macroalgal biomass by invertebrate grazers may contribute towards explaining why the benthic microalgae in stromatolite pools are not competitively replaced by macroalgae. Additionally, top-down forces from predators might act to restrict the grazer biomass to a level at which it is not reliant on stromatolite microalgae as a food resource compared to pool macroalgae. The high proportion of macrofauna in predator/omnivore diets certainly suggests that this is an important food resource for organisms at the top of the food-web. In many intertidal habitats^43^,^44^, as well as some microbial habitats where metazoans co-occur^22^, fish or other predators play an important role in terms of restricting grazer abundance.

Although the relative positions of trophic levels within each site-specific food-web were similar, there was a clear pattern of δ^15^N enrichment between sampling locations from Cape Recife to Seaview. This site variability is likely a function of known differences between sites in terms of nutrient input, particularly DIN^25^,^27^,^28^. Anthropogenic sources of nitrogen (urban waste) are thought to account for the site-specific variability because of different levels of residential occupation^28^. These results therefore further validate the mechanism for the site-specific gradient of DIN input, as anthropogenic pollution is a known, and well-documented, vector for δ^15^N enrichment^45^,^46^. Variability amongst consumer δ^15^N signatures can be used effectively to monitor nitrogen inputs into estuarine systems^47^, and this study therefore provides the first baseline for these SI levels in South African stromatolite systems. Although additional nutrient inputs can affect overall trophic structure^48^, this has not been observed in the stromatolite systems yet, perhaps because the nutrient levels remain below a threshold which might change community composition (*sensu* Forbes *et al*.^49^).

### Conclusions and future recommendations

The results presented here further demonstrate that the peritidal stromatolites along the South African coastline form under an inter-linked balance of drivers and pressures. The surprising occurrence of metazoans together with lithifying microbial mats appears to be due to the exclusion of most typical and potentially-destructive intertidal organisms that cannot tolerate the frequent salinity regime shifts^28^ as well as the microrefugia benefit derived from the stromatolite matrix by the metazoans^20^, which may also buffer against salinity fluctuations within this peritidal zone^28^. These patterns of marine-freshwater cycles also affect the macroalgal community^25^, with biomass being further controlled by metazoan grazers (this study). Physico-chemical forces allow the stromatolites to thrive at these locations, such as the calcium carbonate input which is necessary during the microbial mineralisation process^11^,^26^, and the optimal nutrient convergence conditions within the main barrage pool in terms of DIN and DIP^28^. This nutrient regime contributes towards the high benthic microalgal biomass that builds the stromatolites^27^, but does not support an abundant phytoplankton community because of the low water retention time within the pools^29^.

A balanced system such as this is likely consistent amongst modern microbialite ecosystems. For example, Garcia-Pichel *et al*.^21^ demonstrated that spherical stromatolites (oncolites) forming in the Rio Mesquites, Mexico, are able to persist because of the balance between high calcium carbonate levels, that enable microbial accretion, and the slower rate of bioerosion by grazing gastropods. Additionally, predatory fish restrict metazoan grazer biomass^22^ and nutrient availability affects the calcification potential of the benthic microalgal community^50^. Threats which may disrupt the balance of these ecosystems could place the rare stromatolite formations at risk. It is therefore unsurprising that some microbialites have been formally protected^13^,^49^ or their protection advocated^25^, in terms of factors such as water requirements.

While the results presented here are informative, several recommendations should be made for future work. Collections from additional seasons would determine whether consumer and source signatures are consistent. Although SI analysis is a measure of long-term dietary consumption^30^,^31^, the SI signature of short-lived species, such as invertebrates, may change depending on seasonal resource availability. This may also provide additional and sufficient material for those groups, such as the chironomids, where multi-site samples were pooled due to mass constraints, which would thereby tease out potential SI signature variation between the combined species or samples. Furthermore, mixing models will always generate a solution to consumer diet based on the sources provided^51^. While every effort was made to verify that only ecologically-relevant sources were included in mixing models in this study, stomach content dietary analyses would validate these decisions. For organisms at the apex of the stromatolite food-web, especially the mobile brachyurans which can move beyond the stromatolite pools, some sources may be missed during collections, particularly those of an ephemeral nature such as carcasses of marine organisms washed into the pools during storm surges. Future studies would benefit from quantification of these sources of uncertainty through dietary studies and repeated seasonal sampling in order to validate the observations presented here.

## Methods

### Study site

Stromatolites form along the South African coastline at areas where groundwater that is rich in calcium carbonate interacts with marine water at the upper intertidal to supratidal zone^26^, currently known from over 500 locations^25^. Three representative stromatolite localities have been investigated previously^20^,^25^,^27^,^28^,^29^ at Cape Recife (site A; 34°02′42.13′S, 25°34′07.50”E), Schoenmakerskop (site B; 34°02′28.23”S, 25°32′18.60”E) and Seaview (site C; 34°01′03.16”S, 25°21′56.48”E), southwest of Port Elizabeth, located within the biodiverse Agulhas bioregion^52^. As such, these were the locations for sample collection during the current study. This region of the South African coastline is exposed to high-energy wave and storm conditions, with a microtidal (≤2.0 m), diurnal tidal regime^53^.

The stromatolite pools are characterised by distinct areas of accretion, defined by their position relative to the inflowing groundwater and proximity to the marine high-water mark^25^. The middle, or ‘barrage’^49^, pool (0.3–0.7 m depth) supports the bulk of the stromatolite biomass^27^ and undergoes regular shifts between marine and freshwater conditions, with salinity fluctuating between ~1 to ≥ 30 depending on tidal and storm conditions^28^. Associated sample collections were confined to this middle region, rather than in the upper (landwards) or lower (seawards) pools, which support minimal stromatolite growth. Allochthonous sample collections were also taken from the inflowing stream source and adjacent marine environments.

### Sample collections

Samples were collected during August 2015 at each of the three sites during low tide. To assess basic pool conditions, physico-chemical, nutrient and microalgal biomass/composition parameters were measured in the barrage pool. Nutrient conditions were also recorded from water in the inlet stream and adjacent marine environment. Measurements included: temperature, salinity, turbidity, dissolved oxygen and pH using a YSI 6600-V2 multiprobe (YSI, Yellow Springs, USA); benthic and pelagic microalgal biomass (chlorophyll-*a*) after extraction in 90% acetone and recorded on a Turner 10-AU narrow-band system (Turner Designs, Sunnyvale, USA); *in situ* benthic microalgal composition using a BenthoTorch (bbe Moldaenke GmbH, Schwentinental, Germany); and dissolved inorganic nitrogen (DIN) and phosphorus (DIP). Collection methods and laboratory processing procedures for these parameters are presented elsewhere in detail^27^,^29^.

The following components of the stromatolite community at each site were collected for subsequent carbon and nitrogen SI analysis. Suspended, particulate organic matter (POM) was extracted from the inlet stream, the middle barrage pool and the adjacent ocean by filtering approximately 2 L of water from each source over pre-combusted (450 °C, 5 h) Whatman glass-fibre filters (GF/F; 1 μm). Sediment was collected from benthic grabs at each barrage pool (apart from at Schoenmakerskop, where little to no benthic sediment was supported) and inlet stream, removing obvious shell fragments, macrofauna, or plant matter. Additionally, stromatolite sediment was collected from cores after removing the upper 1.5 cm of the matrix, which contains the actively accreting component^27^. Decaying plant matter (detritus) was hand-collected from the inlet stream. Living macrophytes, grouped according to grasses (monocotyledons) and forbs (dicotyledons), were cut from vegetation associated with each barrage pool and inlet stream. Living macroalgae were hand-collected from the nearby ocean and barrage pool. Microalgae were differentiated according to those forming the stromatolite material, collected by scraping the upper 1–2 mm of the stromatolite matrix^26^, and those at the periphery, scraped off from rocks within the stromatolite pools.

Cores of stromatolite material were excised using a stainless steel corer and rubber mallet^20^ to obtain invertebrate infauna. Epifauna were collected from scrapes of rocks within the barrage pools and using a 1 mm sweep net. Zooplankton was collected by straining at least 100 L of pool water over a 100 μm sieve. Large brachyurans were hand-collected and fishes were caught using sweep nets or small, baited hooks. All faunal samples were cooled rapidly to below zero and stored frozen before laboratory processing. Ethical clearance was granted by the Research Ethics Committee (Animal) at the Nelson Mandela Metropolitan University (Reference: A15-SCI-ZOO-011) and all relevant guidelines and regulations were adhered to during sample collections.

### Sample processing

Tracing organic carbon flow through a food-web requires the removal of the inorganic carbon component^54^,^55^. This is typically achieved by excising and analysing tissue that contains inconsequent amounts of inorganic carbon (such as muscle tissue), removing the structure that has a high amount of inorganic carbon (such as bone or shell), or chemically reacting/dissolving the inorganic carbon through acid treatment. As the latter can have undesirable consequences on nitrogen SI ratios^54^, duplicate samples of each component were analysed for acidified versus non-acidified treatments.

Filters containing POM were first oven-dried (at 60 °C for at least 48 h, the standard procedure for all components in this study) and to one set of duplicate filters, 0.25 N HCl was added dropwise until effervescence had ceased^56^ and thereafter dried as before. Filters were stored in sterilised aluminium foil prior to SI analysis. Sediment was oven-dried before being homogenised to a fine powder with an agate pestle and mortar. Half of each sediment sample was placed in clean 20 ml glass scintillation vials and 1 N HCl was added dropwise until effervescence had ceased^54^,^57^. Acid treatment of samples containing a high proportion of inorganic calcium carbonate, as is the case for stromatolite material, reacts to create hygroscopic calcium chloride crystals. This interferes with the grinding and crushing process and can affect the SI measurement instrumentation deleteriously^57^. To overcome this hindrance, distilled water (triple the volume of the sediment) was added to each sample and then centrifuged, to minimise loss of the sample particles, at 2000*x*g for five minutes. This was repeated three times and the sample was then oven-dried and ground as before. Sediment samples were stored in sterilised 2 ml polypropylene vials prior to SI analysis. Detritus, macrophytes, macroalgae and microalgae were rinsed with distilled water and any epibiont or sediment contaminants removed before oven-drying. These were ground to a fine powder and 3–5 replicate sub-samples (1.0 ± 0.05 mg each) placed in sterilised tin capsules (5 × 9 mm; SÄNTIS Analytical AG, Switzerland) prior to SI analysis. Duplicate quantities of these samples were acidified and rinsed, as for the sediment samples, before weighing into tinfoil capsules.

Infauna and epifauna were sorted under a dissection microscope according to dominant species or taxa identified from the stromatolite ecosystems^20^,^25^, before drying separately. The shells of gastropods were removed and discarded, while muscle tissue from fishes, brachyurans and penaeid shrimps was excised, prior to drying. Tissue with a high lipid-content can skew the meaningful carbon isotope ratio of animals^56^,^58^. As such, the lipids from invertebrates dried whole were extracted following Logan *et al*.^58^. A replicate half of powdered samples from organisms that contain a chitonous exoskeleton (e.g. amphipods and isopods) were acidified drop-wise with 0.25 N HCl until effervescence ceased^55^,^56^. After drying, all samples were powdered and weighed (0.5 ± 0.05 mg) into 3–5 replicate tinfoil capsules. Samples with insufficient weight for SI analysis were pooled from multiple organisms of the same species or taxonomic group. Chironomid samples, in particular, had an especially small dried weight and it was therefore necessary to combine these across sites.

### SI analyses

All samples were processed at iThemba Laboratories (Johannesburg, South Africa) on a Flash HT Plus elemental analyser, coupled to a Delta V Advantage isotope ratio mass spectrometer by a ConFloIV interface (all supplied by ThermoFisher, Bremen, Germany), to determine carbon (^13^C and ^12^C) and nitrogen (^15^N and ^14^N) concentrations and ratios. SI data are represented as the relative (‰) difference between samples and the international standard for C (Pee Dee Belemnite carbonate) and N (atmospheric N_2_):


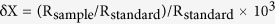


where X is ^13^C or ^15^N and R is the ^13^C:^12^C or ^15^N:^14^N ratio. SI values were corrected against a standard of known concentrations (Merck Gel), which was run after every 12 or 24 unknown samples. The 1σ precision of all standards (*n* = 62) was ±0.10‰ and ±0.08‰ for C and N, respectively.

### Data analysis

Some quantities of sample material were insufficient for both acidified and non-acidified treatments, due to constraints related to the destructive nature of sample collection. This was especially apparent for the malacostracans (amphipods, isopods and tanaids). An acidification conversion relationship was therefore calculated using those samples that had sufficient material for both treatments (Supplementary Fig. S1). This well-fitted relationship (R^2^ = 0.889 to 0.982) was then used to determine the decarbonated δ^13^C value for non-acidified samples (*sensu Bonn & Rounds*^59^).

All data were analysed in R^60^ using the ‘nlme’, ‘SIBER’ and ‘MixSIAR’ packages^61^,^62^,^63^. Sources and consumers collected were assigned to relative trophic guilds based upon known life-history characteristics. The isotopic niche space of these guilds were compared between sites using Bayesian ellipses^63^, as well as niche community metrics such as the total area (TA) of the convex hull encompassed by all isotope values^64^. Specifically, the standard ellipse area (SEA) and its corrected value (SEAc) were calculated for each trophic guild. The SEAc is a robust measure of community structure, resistant to sample size limitations^63^. To assess the variability amongst organic matter source signatures within the stromatolite pools, the δ^13^C signatures were compared using a linear mixed-effects modelling (LMM) approach^65^. This assessed the optimal residual variability for potential random effects (nominal variables: sampling sites and organic matter sources) before testing the significance of site and organic matter sources as fixed effects for the response variable, δ^13^C^65^,^66^. Similarly, the relative difference between infaunal and epifaunal consumers in terms of δ^13^C was assessed using an LMM, testing for site, location (epifauna or infauna), and taxonomic groups as possible random effects, with location being the fixed effect. Normality of residuals and homogeneity of variance across all fixed effects were assessed for LMMs to meet model assumptions^65^.

Knowledge of SI concentrations within consumers relative to food sources have allowed ecologists for just over a decade to mathematically establish diet composition due to known pathways of trophic fractionation within food-webs^67^,^68^. Termed ‘mixing models’, rapid recent development in both sophistication and application^32^ has led to statistical tools that can assign diet composition with greater certainty while accounting for variability associated with food sources and availability, trophic fractionation, and nutrient composition, amongst others^51^. Seminal statistical tools for mixing models, including IsoSource^67^,^68^, MixSIR^69^, and SIAR^70^, have since been compiled in a collaborative, centralised, freely available and powerful software package, MixSIAR, which uses Bayesian statistics^62^. One of the critical advantages of this is that it accounts for uncertainty and prior information in source partitioning^69^,^71^. This software was used here to determine diet composition of the dominant stromatolite infauna and epifauna, as well as the higher-level predators and scavengers.

Organic matter sources were selected from those that are ecologically relevant to the consumer species. All sources that were statistically indistinguishable in terms of SI composition, and which were ecologically similar, were combined *a priori*^35^. For consumers with many potential sources, particularly omnivores, individual sources were aggregated *a posteriori* into ecologically-relevant groupings by summing the posterior distribution probabilities^35^,^51^. Additionally for omnivores, the variability amongst sources in terms of C and N concentrations (for plants or algae compared to animal material) was accounted for using a source concentration structure specified in the mixing model^72^. Substantial uncertainty in SI analyses relates to the dietary assimilation or fractionation of isotopes from sources to consumers^51^. As MixSIAR can account for some degree of this within a residual error term^62^,^70^, a conservative estimate of trophic fractionation for δ^13^C and δ^15^N was specified (1.0 ± 0.25 and 2.0 ± 0.5‰, respectively), based upon published recommendations^73^,^74^ and site-specific knowledge of baseline organic matter sources relative to primary consumers^75^ obtained during this study. Diet composition was assessed as the posterior probability distributions of the various food sources. Taxonomic group (random effect: primary consumers; fixed effect: brachyurans; not included for shrimps or fish as only single species were assessed) was nested within sampling site (random effect for all models) as a hierarchical structure^76^.

Data are presented as mean ± SD, unless otherwise indicated, and an *a priori* significance level of α = 0.05 was specified.

## Additional Information

**How to cite this article**: Rishworth, G. M. *et al*. Non-reliance of metazoans on stromatolite-forming microbial mats as a food resource. *Sci. Rep.* **7**, 42614; doi: 10.1038/srep42614 (2017).

**Publisher's note:** Springer Nature remains neutral with regard to jurisdictional claims in published maps and institutional affiliations.

## Supplementary Material

Supplementary Information

## Figures and Tables

**Table 1:** Summary of physico-chemical properties for surface and benthic barrage pool water measured at the three stromatolite sites at the time of sample collections during August 2015 (winter), recorded from three stabilised measurements.

*Winter*		Temp (°C)	Sal	Turb (NTU)	DO (mg.l^−1^)	pH	DIN (μM) I; P; O	DIP (μM) I; P; O	Pelagic chl-*a* (mg.m^−3^)	Benthic chl-*a* (mg.m^−2^) Cy;Ch;Di (%)
A	Surface	17.3	1.4	0	12.5	8.6	82; 71; 9	0.0; 0.0; 0.2	4.0 ± 0.2	517.4 ± 64.5
	Bottom	17.0	1.6	0	11.0	8.3				54; 0; 46
B	Surface	14.9	1.2	0	10.7	8.9	424; 289; 14	0.0; 0.0; 0.0	1.2 ± 0.1	576.9 ± 81.3
	Bottom	16.1	4.9	0	11.2	8.8				51; 0; 49
C	Surface	17.0	1.4	0	8.9	7.7	462; 321; 9	0.1; 0.4; 0.2	2.2 ± 0.3	1442.9 ± 171.3
	Bottom	18.8	21.6	0	6.1	7.9				47; 0; 53

Temp (*Temperature*); Sal (*Salinity*); Turb (*Turbidity*); DO (*Dissolved Oxygen*); DIN (*Dissolved Inorganic Nitrogen*); DIP (*Dissolved Inorganic Phosphorus*); I (*Inlet*); P (*Pool*); O (*Ocean*); Cy (*Cyanophyta*); Ch (*Chlorophyta*); Di (*Bacillariophyta*, mostly *Diatoms*). Also indicated are nutrient concentrations for source (inlet and ocean) and pool water as well as benthic and pelagic microalgal biomass within each barrage pool (±SD), with the latter also reflecting proportional contributions by cyanobacteria, chlorophytes and diatoms. Nutrient data were taken from Rishworth *et al*.^28^.

**Table 2:** Summary of the Bayesian isotope niche metrics for the trophic guilds at the three stromatolite sites along the South African coastline during August 2015.

Site	Trophic guild	Standard ellipse area (‰^2^)			SEAc				
		*Mode*	*50%*	*95%*					
A	Organic matter source	32.4	26.8–39.0	17.2–57.2	42.0				
	Grazer/collector	4.4	3.5–5.5	2.2–9.2	5.8				
	Predator/scavenger	3.8	2.8–5.0	1.1–9.4	5.1				
	*Overall TA*	*7.4*	*6.6–8.5*	*4.6–10.6*					
B	Organic matter source	21.8	18.4–26.4	12.4–38.2	45.7				
	Grazer/collector	5.1	4.2–6.4	2.8–9.5	9.3				
	Predator/scavenger	18.4	13.1–24.7	4.7–50.8	18.1				
	*Overall TA*	*1.5*	*0.7–2.3*	*−0.2–3.9*					
C	Organic matter source	29.6	25.3–37.0	17.4–53.1	43.1				
	Grazer/collector	9.0	7.4–11.4	5.0–17.9	9.3				
	Predator/scavenger	5.7	4.3–7.3	2.1–13.2	6.6				
	*Overall TA*	*3.2*	*2.7–3.9*	*1.5–5.2*					

The mode Standard Ellipse Area (SEA) is shown with associated 50% and 95% quantile distributions. The corrected SEA (SEAc), which reflects the mean SEA after accounting for small sample sizes, for each trophic guild and the mode total area of the convex hull (TA) encompassed by all isotope data points at each site are also presented.

**Table 3:** Mean (±SD) δ^13^C across all three stromatolite sampling locations during August 2015 for the organic matter sources collected within the main stromatolite pools.

	δ^13^C	GLS model			
	Mean (±SD) ‰	C (±SE)	t	P	var
Stromatolite microalgae	−17.6 (±1.4)	*			0.11
Benthic SOM	−20.4 (±0.4)	−2.5 (±0.4)	−5.9	<0.001	0.00
Pool macroalgae	−25.3 (±5.9)	−8.0 (±3.8)	−2.1	0.06	1.00
Pool microalgae	−16.7 (±3.9)	0.6 (±2.2)	0.3	0.78	0.59
Pool POM	−20.5 (±0.8)	−3.0 (±0.5)	−6.5	<0.001	0.06
Stromatolite SOM	−24.0 (±0.2)	−6.6 (±0.6)	−11.8	<0.001	0.10
Cape Recife	−22.3 (±4.4)	*			
Schoenmakerskop	−19.8 (±5.6)	0.7 (±0.4)	2.1	0.07	
Seaview	−20.0 (±2.6)	−0.6 (±0.0)	>100	<0.001	

*‘Stromatolite microalgae’ and ‘Cape Recife’ are the reference values used in the GLS analysis. SOM (*sediment organic matter*); POM (*particulate organic matter*). Also shown are the results of a generalised least squares (GLS) analysis of δ^13^C, with the stromatolite pool organic matter sources and sampling sites as model predictor variables. Different variance structures (var) according to organic matter source accounted for within the GLS model, as well as the coefficient (C) and test significance of each predictor, are also indicated.
