# Supplementary material for: Non-reliance of metazoans on stromatolite-forming microbial mats as a food resource
Source: Sci Rep. 2017 Feb 16;7:42614. doi: 10.1038/srep42614 (PMC5311985; doi:10.1038/srep42614)
Supplement: Supplementary Information [file srep42614-s1.pdf]

Non-reliance of metazoans on stromatolite-forming microbial mats as a food resource

Gavin M. Rishworth[1,*], Renzo Perissinotto[1], Matthew S. Bird[1], Nadine A. Strydom[2], Nasreen Peer[1], Nelson A.F. Miranda[1], Jacqueline L. Raw[1]

[1]DST/NRF Research Chair in Shallow Water Ecosystems, Nelson Mandela Metropolitan University, Port Elizabeth 6031, South Africa

[2]Department of Zoology, Nelson Mandela Metropolitan University, Port Elizabeth 6031, South Africa

*corresponding author: gavin.rishworth@gmail.com

Supplementary material

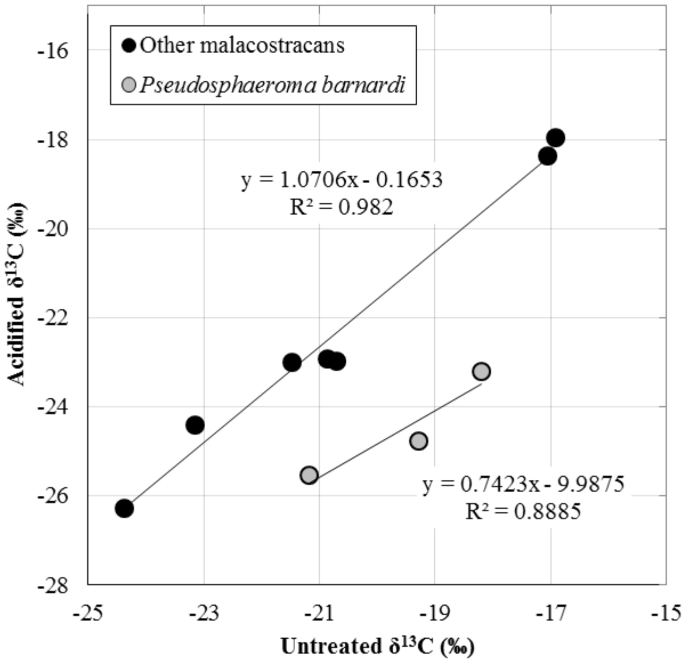

Figure S1 Linear relationships between malacostracan samples that had both acidified and non-acidified replicates. The fit of these relationships (R^2) are also shown. Samples are separated by *Pseudosphaeroma barnardi* (with a more calcareous exoskeleton) and 'other malacostracans', which include *Melita zeylanica*, *Orchestia rectipalma* and *Ectias angusta*.

Table S1. Summary statistics of the relative dietary source proportional contributions for consumer groups (macroinvertebrates – see Figure 3, gobiids, shrimps and brachyurans) sampled from three stromatolite locations during August 2015. Diets were assessed using $\delta^{13}C$ and $\delta^{15}N$ isotopes as biotracers within a Bayesian mixing model, fitted using MixSIAR[62]. Macroinvertebrates were assessed with taxonomic groups nested as a random effect within site. All other consumer groups only included site as a random effect, with the two brachyuran species assessed independently as fixed effects. Data are presented as mean ± SD as well as the 50% (median) and 95% quantiles.

Consumer	Source	Mean	±	SD	Median	95% quantile		
Macroinvertebrates - infauna and epifauna	Inlet OM	15%	±	11%	12%	1%	–	42%
	Ocean macroalgae	11%	±	10%	9%	0%	–	36%
	Ocean POM	7%	±	7%	4%	0%	–	27%
	Pool macroalgae	26%	±	15%	24%	3%	–	58%
	Pool microalgae	11%	±	10%	8%	0%	–	36%
	Pool OM	10%	±	10%	7%	0%	–	36%
	Stromatolite microalgae	10%	±	10%	7%	0%	–	37%
	Stromatolite SOM	11%	±	10%	8%	0%	–	37%
Coryogalops sordidus - gobiid	Chironomids	8%	±	8%	6%	0%	–	28%
	Gastropods	8%	±	8%	6%	0%	–	30%
	Malacostracans	11%	±	9%	8%	0%	–	34%
	Marine primary sources	17%	±	11%	15%	2%	–	43%
	Polychaetes	11%	±	9%	9%	1%	–	35%
	Pool primary sources	34%	±	14%	33%	10%	–	62%
	Shrimps	12%	±	9%	9%	1%	–	35%
Palaemon peringueyi - shrimp	Chironomids	21%	±	15%	19%	1%	–	55%
	Gastropods	19%	±	12%	17%	1%	–	45%
	Gobiids	26%	±	13%	27%	2%	–	53%
	Malacostracans	16%	±	13%	13%	1%	–	48%
	Polychaetes	18%	±	13%	16%	1%	–	49%
Cyclograpsus punctatus - brachyuran	Chironomids	10%	±	8%	9%	0%	–	30%
	Inlet primary sources	12%	±	8%	11%	2%	–	32%
	Gastropods	6%	±	6%	4%	0%	–	21%
	Gobiids	6%	±	5%	4%	0%	–	20%
	Malacostracans	8%	±	7%	6%	0%	–	26%
	Marine primary sources	13%	±	8%	12%	2%	–	33%
	Polychaetes	5%	±	5%	4%	0%	–	20%
	Pool primary sources	31%	±	11%	30%	12%	–	56%
	Shrimps	8%	±	6%	7%	0%	–	24%
Potamonautes perlatus - brachyuran	Chironomids	10%	±	12%	6%	0%	–	44%
	Inlet primary sources	16%	±	16%	11%	1%	–	59%
	Gastropods	4%	±	6%	2%	0%	–	22%
	Gobiids	6%	±	7%	3%	0%	–	25%
	Malacostracans	10%	±	11%	5%	0%	–	42%
	Marine primary sources	14%	±	13%	9%	1%	–	48%
	Polychaetes	5%	±	7%	2%	0%	–	25%
	Pool primary sources	29%	±	17%	26%	6%	–	69%
	Shrimps	7%	±	8%	4%	0%	–	29%

- Chironomid sources were taken as a combined $\delta^{13}C$ and $\delta^{15}N$ signatures across sites, while gastropods were only collected from stromatolite pools at Schoenmakerskop, but their signature was approximated for all gastropods across sites.
- For omnivorous species (gobiids and brachyurans), primary sources were aggregated *a posteriori* from Bayesian mixing model posterior distributions. *Inlet primary sources* – detritus and inlet OM; *Marine primary sources* – ocean macroalgae and ocean POM; *Pool primary sources* – pool macroalgae, pool microalgae, pool OM, stromatolite microalgae and stromatolite SOM.
- SOM (*sediment organic matter*); POM (*particulate organic matter*); OM (*organic matter as both SOM and POM*)
